# A long, strange trip: Ketamine treatment in psychiatry

**DOI:** 10.1177/02698811251379393

**Published:** 2025-10-27

**Authors:** David J. Nutt, Celia Morgan, David Erritzoe, Kyle T. Greenway, Allan H. Young

**Affiliations:** 1 Division of Psychiatry, Imperial College London and Solvonis, UK; 2 Department of Psychology, University of Exeter, UK; 3 Division of Psychiatry, Imperial College London, UK; 4 Lady Davis Institute, Jewish General Hospital, Montreal, QC, Canada; 5 Department of Psychiatry, McGill University, Montreal, QC, Canada

## Introduction

It is 60 years since ketamine was first tested in humans as an alternative but safer anaesthetic to PCP (phencyclidine) (Corssen and Domino 1966). It became widely used as the “buddy drug” for combatants in the Vietnam War: because of its great safety margin, it could be injected into wounded personnel by their buddies to relieve pain before the arrival of the medical team.

Because of ketamine’s great safety margin in terms of respiratory depression, plus its analgesic properties, it is widely used all over the world in places where mechanically ventilated anaesthesia for surgery is often not feasible, especially sub-Saharan Africa. Hence, ketamine has been on the World Health Organisation’s essential medicine’s list since 1985.

Note − in this article, we use ketamine to refer to the anaesthetic/analgesic product racemic ketamine. Where we comment on specific enantiomers, we use the names esketamine or arketamine for the S and R enantiomers, respectively.

### 1970s–1990s Early clinical work with ketamine using its dissociative effects

The first clinical work with ketamine in psychiatric disorders started in the 1970s with clinicians utilising the psychoactive effects in psychiatric patients for therapeutic benefits. One pioneer in this field was the Mexican psychiatrist Roquet who added ketamine treatment technique he called “psychosynthesis” for a range of different psychiatric disorders (see Kolp et al., 2014). Ketamine (1.5 mg/kg IM) and other psychedelic drugs were combined with psychotherapeutic techniques in order to “. . .contribute to the cathartic process,” as well as “. . .enhance personal growth and self-awareness” (Kolp et al., 2014). Patient with neurosis, personality disorders, and a subgroup of patients with psychosis were treated (see Walsh et al., 2022) The sessions took place overnight and involved sensory overload of patients with loud music plus some violent and sexually explicit films. The high doses of the ketamine and other psychedelic drugs plus the sleep deprivation were designed to break down entrenched mental processes. Roquet claimed many thousands of patients were helped with an 85% success rate and no one having enduring harms https://chacruna.net/salvador-roquet-remembered/).

Other early reports described using ketamine (instead of barbiturates) as an “abreactive agent” for patients with anxiety, depression, phobias, and obsessive-compulsive disorders (Khorramzadeh and Lofty, 1973). But abreaction as a psychological technique was in rapid decline and ketamine with it.

The state of altered consciousness produced by ketamine underpinned the early work with ketamine for addictions, which Krupitsky began in the USSR in the 1980s. Initially, ketamine was used as aversive therapy to induce strong negative emotions towards alcohol so deterring use (Krupitsky et al., 1992). They then realised there was potential to use psychedelic doses of ketamine to potentiate psychotherapy, developing a randomised clinical trial (RCT) of ketamine plus psychedelic psychotherapy for alcohol dependence (Krupitsky and Grinenko, 1997). Results were impressive with an abstinence rate in the ketamine group of 66% at 1 year compared with only 24% in the control group (Krupitsky and Grinenko, 1997). The Krupitsky team then conducted an RCT using the same approach in heroin addiction, comparing a high, psychedelic dose of ketamine (2.0 mg/kg IM) with a low, sub-psychedelic dose of (0.2 mg/kg IM) (Krupitsky et al., 2002). Both groups received 10 hours of preparatory psychotherapy before the ketamine dose and 5 hours after. In both groups, immediate reductions in craving were found. This benefit was maintained over 24 months for the high dose group only and likely contributed to their maintaining greater abstinence from heroin over that period (Krupitsky et al., 2002). This hugely innovative work was terminated in 2002 when, after the collapse of the Soviet Union, ketamine was removed from medical use in an attempt to reduce recreational use.

Other early explorations of ketamine’s psychoactive effects and their clinical consequences took place in various places around the world in the first decades following its discovery (Diep et al., 2025). In London in 1972, Barbara Collier compared the experiences of 131 patients receiving ketamine anaesthesia for minor surgical procedures to 80 patients receiving other anaesthetic agents and found that ketamine was unique in inducing “floating” feelings and vivid visual hallucinations with bizarre, dream-like, or even transcendental qualities, similar to those induced by serotonergic psychedelics and sensory deprivation (Collier, 1972). Interestingly, these experiences were recalled with much greater clarity by subjects questioned immediately post-anaesthesia rather than only the following day. Along these same lines, a 1981 study by New York anaesthetists (Sklar et al., 1981) demonstrated that a simple pre-anaesthesia suggestion – that ketamine would induce pleasant dream-like experiences, which were under patients’ control – could psychologically “abolish” the otherwise commonly reported distressing experiences induced by ketamine. Such studies added to the growing awareness that, even in acute medical contexts, ketamine’s potent psychoactive effects are shaped by non-pharmacological contextual factors much like the serotonergic psychedelics. Similarly, in psychiatric contexts, a remarkable 1988 Danish study by Karl Hansen demonstrated that subanaesthetic ketamine could yield meaningful and psychologically beneficial psychedelic experiences when adequate attention is paid to “sets and settings,” such as the provision of music and psychological support. Indeed, much like the serotonergic psychedelics, the authors concluded that ketamine could improve psychotherapeutic treatments and facilitate mental health practitioner training.

More recent clinical trials of ketamine treatment for alcohol and substance use disorders have built on the pioneering psychedelic work of Krupitsky and others but with more rigorous research methods including random assignment to treatment, blinding of participants and researchers, as well as placebo (active/saline) and psychological treatment control to address some of the methodological limitations of earlier research (Dakwar et al., 2014, 2017, 2020; Grabski et al., 2022). Compared with the benzodiazepine lorazepam, a single ketamine infusion (0.41 mg/kg) led to significant reduction in motivation to use cocaine and cue-induced craving at 24 hours, which were further enhanced by the subsequent ketamine infusion (0.71 mg/kg) (Dakwar et al., 2014). In a larger study, Dakwar et al. (2017) demonstrated that a single ketamine infusion (0.71 mg/kg) led to significant reductions in cocaine self-administration at 28 hours compared with the benzodiazepine midazolam control (0.025 mg/kg) among a population with cocaine use disorder.

## 1980s − Mechanistic breakthrough: Ketamine is an N-methyl-D-aspartate receptor antagonist

The pharmacology of ketamine eventually became understood in the 1980s. It followed the discovery by Watkins and Evans (2006) at Bristol University in the 1970s that glutamate was the key excitatory neurotransmitter in the brain, and that this acted in part through what we now call the N-methyl-D-aspartate (NMDA) receptor. They also demonstrated the remarkable role of magnesium ions in blocking the ion channel of which the receptor is a part, so limiting brain excitation. A little later, in the early 1980s, Lodge et al. (1982) found that ketamine blocked the NMDA receptor, which explained its anaesthetic, analgesic, and dissociative actions.

Because ketamine was easily accessible in a medical formulation, it was (and still is) the easiest NMDA receptor antagonist to use for the study of these receptors in humans. In the late 1980s, the Nutt group at Bristol University along with Watkins and Evans began to explore the use of a ketamine challenge in humans as a measure of NMDA receptor function in people with alcoholism. The theory was that the magnesium deficiency commonly seen in dependent drinkers would result in less tonic block of the receptor so leading to the hyperexcitable state of alcohol withdrawal (Nutt and Glue, 1990). We developed a ketamine challenge test using I.M. dosing with the outcome measures being subjective changes and slowing of saccadic eye movement velocity. The prediction was that the effects of ketamine would be reduced in the alcohol dependent population due to their being in a state of NMDA hyper-function from depleted magnesium. However, in the pilot studies with healthy volunteers, we found both the subjective and physiological effects of ketamine to be very variable, meaning any study would have required large numbers of subject for statistical power. Also, some participants said they found ketamine quite euphoric and were keen to be given it again. For these reasons, we decided to terminate the study.

Similar euphoric effects of ketamine were found by the Yale group of Charney and Krystal who had started to use ketamine in human volunteer studies as a model for psychosis (Krystal et al., 1994). They then moved on to use ketamine to probe a possible role of glutamate receptor changes in depression based on the work of Skolnick who had shown in rodents that several other NMDA antagonists had antidepressant-like effects (Trullas and Skolnick, 1990). This direction proved very exciting – see below.

## 2000s − Ketamine for depression: “Liquid ElectroConvulsive Therapy (ECT)”

As early as 1975 pre-clinical animal research had suggested that ketamine might have antidepressant-like properties (Sofia and Harakal, 1975) but the first controlled depression ketamine study came out of the Yale group’s interest in glutamate receptor function. Berman et al. (2000) reported a study of 14 patients with major depression who were randomised to receive either a single dose of ketamine (0.5 mg/kg intravenous (IV)) or saline. By day 3, the ketamine group reported significant reductions in depression scores compared with the placebo group. These promising findings were followed up in a larger study in which 18 patients with treatment-resistant depression were given either a single IV infusion of ketamine (0.5 mg/kg) or IV saline in a crossover design (Zarate et al., 2006). The ketamine group reported significant reductions in their depression as early as 2 hours with a peak at day 3 and still some significant effect at 1 week. Later, the same team also demonstrated that ketamine could reduce suicidal ideation in similar treatment-resistant depression patients (DiazGranados et al., 2010a) as well as lift depressive symptoms, also in patients with bipolar disorder (Diazgranados et al., 2010b; Zarate et al., 2012).

Since then, ketamine has been tested against major and treatment-resistant depression in at least 25 research studies (see Price et al., 2022). These results show that ketamine consistently produces better outcomes compared with placebo, especially in more treatment-resistant patients, as defined by a greater number of failed prior treatments.

As the current best treatment for treatment-resistant depression is ECT, it is not surprising, there have been comparative studies of these two modalities. In a Swedish study of 186 inpatients up to 12 doses of IV ketamine were given over 6 weeks and outcomes compared with standard ECT treatment (Ekstrand et al., 2022). Though ECT was more efficacious in producing remission ketamine performed well, and the authors concluded it should be considered an acceptable alternative to ECT. A similar head-to-head multi-site study was conducted in the USA with 403 treatment-resistant depression patients (Anand et al., 2023). Ketamine (0.5 mg/kg IV) was given twice a week for 3 weeks and compared with ECT three times a week for 3 weeks. In this study, response rates to ketamine were numerically better than those of ECT.

Despite this strong evidence base, ketamine is not an approved medicine for depression (or any indication other than anaesthesia and pain). This is probably because no pharma company believes they could obtain and protect a patent for its use in these indications. However, because of its obvious utility in this extensive data set, ketamine is now widely used off-license in many clinics in the United States, Europe, and some other countries for depression, especially where other treatments have failed (Caddy et al., 2015; Jelen et al., 2024). But for depression ketamine is often administered by anaesthetists rather than by psychiatrists, sometimes with little or no psychiatric oversight. In this use, mostly via the IV route, ketamine is often given in the same clinical facilities as is ECT, hence the colloquial name “liquid ECT.”

In many of these treatment centres, ketamine is given typically twice weekly to produce an antidepressant response, then less frequently as a maintenance therapy, sometimes with at-home use of oral ketamine as lozenges or liquid. In this way, ketamine is seen as a prophylactic relapse-preventing treatment like lithium or lamotrigine (Maruki et al., 2022).

The dual efficacy of ketamine as an anaesthetic and antidepressant raised the exciting possibility that using it as the anaesthetic for ECT therapy might enhance clinical outcomes via complementary antidepressant mechanisms. Several groups have explored this question, for example, in the KANECT study (Fernie et al., 2017), clinical outcomes were compared from one patient group given ECT under IV ketamine anaesthesia (2 mg/kg) with another group given ECT under the usual propofol anaesthesia. Anderson et al. (2017) took a different approach using a lower dose of ketamine (IV infusion at a dose of 0.5 mg/kg) given just before the ECT to explore if it could reduce cognitive deficits from ECT and augment antidepressant efficacy. Neither study found evidence for either of the theorised benefits, perhaps because ketamine and ECT share downstream mechanisms (see later), but the ketamine dosing was well tolerated. Another question to explore is whether ketamine can maintain efficacy after ECT, so avoiding repeated seizure induction, though from the comparative data mentioned above, it might well replace ECT for some patients.

## 2010s − Esketamine

As with many racemic drugs individual enantiomers possess similar, possibly better pharmacological profiles, and this can allow single enantiomers to be patented as novel agents (Nutt and Feetam, 2010). In this tradition, esketamine was approved as an anaesthetic in the 1990s (Himmelseher and Pfenninger, 1998) and is used as such in several European countries. In the USA, Janssen began a research programme using esketamine for depression that culminated in an approval for treatment-resistant depression in 2019 (Jelen et al., 2021; Popova et al., 2019). The treatment method and treatment model differed from that used with ketamine. Esketamine (Spravato) is given intra-nasally rather than by injection, and the current indication is as an adjunctive agent for patients who have not had an adequate response to a course of standard antidepressant medicine (though evidence of efficacy as a monotherapy is now published Janik et al., 2025) Efficacy has been shown in acute treatment trails and maintained with continued treatments over 6 months (Zaki et al., 2023) and compared favourably with a leading current maintenance treatment quetiapine (Reif et al., 2023).

## 2020s − Ketamine plus psychotherapy

The early studies of ketamine in addiction, as with the large number of depression trials, gave ketamine infusions without psychotherapy. But recently, this approach has been challenged by the resurgence of the older therapeutic model – ketamine-assisted psychotherapy. This has been applied to ketamine both in the treatment of addictions and depression (Walsh et al., 2022).

Addictions: Several psychotherapy modalities have been added to ketamine treatments: these include third-wave cognitive behavioural approaches such as motivational enhancement therapy, which aims to increase individual’s motivation to remain abstinent (Dakwar et al., 2020) and mindfulness-based relapse prevention (MBRP; Gent et al., 2024; Grabski et al., 2022). In a seminal study of people with alcohol dependence, MBRP psychotherapy was provided after each of three ketamine treatments a week apart (Grabski et al., 2022). This was one arm of a sophisticated four-arm design study whereby 96 patients were randomised to four equal groups, two of which got ketamine (0.8 mg/kg IV over 40 minutes) and two placebo (IV saline). Within each of these groups, one was given MBRP, whereas the other received alcohol education. The ketamine MBRP group did best with over 80% days abstinent over the following 6 months. The ketamine education group was ranked second followed by the placebo MBRP with the placebo education group showing the least effect (Grabski et al., 2022). Overall, ketamine treatment was significantly better than placebo. This positive result with enduring benefits of just three ketamine/MBRP sessions led to the UK NIHR funding a phase 3 two-arm trial in collaboration with Solvonis Therapeutics (https://solvonis.com).

The results of these trials support the combination of ketamine with a psychological treatment, as the observed therapeutic effects were maintained for longer follow-up periods compared to the mostly transient effects of ketamine infusions alone (Dakwar et al., 2014, 2017). Other addictions seem to respond similarly. The first clinical trial of ketamine treatment for cannabis use disorders was reported in 2021 (Azhari et al., 2021), where integration of ketamine with psychological treatment with MBRP was, at 1 week, associated with reductions in cannabis use and this effect was maintained at the study end of 6 weeks.

## Depression

Here, the potential value of adding psychotherapy to ketamine treatment has been rather longer in coming than for addiction. In part, this reflects the original view of ketamine that it was just another physical treatment like ECT, not least because it was usually dosed by an anaesthetist in ECT suites. As well as the empirical findings of efficacy described already, ketamine use was also justified in that it shared several brain mechanisms with ECT, such as glutamatergic (Pfleiderer et al., 2003) and serotonergic effects (e.g. Evans et al., 1976). The short duration of therapeutic action after each session was also similar to that of ECT, so justifying repeated doses. This view was consolidated by the development programme of esketamine where eventually a treatment regime of many months’ duration has been recommended (Zaki et al., 2023).

In this framework, the dissociative effects of ketamine and esketamine are generally seen as a nuisance adverse effect, requiring supervision for several hours to avoid disturbed behaviours. However, recently, this view has been challenged, largely due to a rediscovery of the earlier studies and the growing work in addiction mentioned above: here just a few administrations coupled with post-trip psychotherapy produced enduring outcomes, not dissimilar to those found following just one or two doses of serotonergic psychedelics (for review see Nutt et al., 2024). Ketamine can produce very similar subjective (Hansen et al., 1988; Studerus et al., 2010) and brain effects (Diep et al., 2025; Pallavicini et al., 2019) to the serotonergic psychedelics. In particular, both produce an entropic state that seems to disrupt the ruminative cognitions of depression (Carhart-Harris and Nutt, 2017). Moreover, both ketamine (Kopelman et al., 2023) and serotonergic psychedelics (Aleksandrova and Phillips, 2021) induce neuroplasticity, which plausibly allows new positive self-learnings that psychotherapy induces to become established.

Several academic groups have begun to trial ketamine-psychotherapy treatments with significant success in depression, particularly the Montreal group (Garel et al., 2023: Greenway et al., 2024) and also in pain disorders (reviewed in Drozdz et al., 2022). Such approaches have also been approved in the United Kingdom for non-NHS providers, most notably the Awakn clinics in Bristol and London (https://www.cqc.org.uk/location/1-10123727822/inspection-summary).

Although no placebo-controlled RCT has evaluated ketamine-psychotherapy for depression, a recent RCT provides some evidence for the combined therapy. In the Music for Subanesthetic Ketamine trial, 32 severely treatment-resistant patients with depression were randomised to receive music or mindfulness-based support during a standard course of subanaesthetic ketamine infusions paired with psychotherapy (Greenway et al., 2025). This trial found large benefits for depression, anxiety, and suicidality in both groups on par with previous trials that administered ketamine without psychological support but with notably superior persistence, to at least 1 month following the final dose. Additionally, patients receiving ketamine in the study’s psychedelic-like context reported highly psychedelic experiences, with elevated scores on the Mystical Experience Questionnaire and Emotional Breakthrough Inventory of similar magnitude to those seen in recent trials of psilocybin. In this trial, mystical experiences were strongly related to immediate and sustained psychiatric benefits at the group level and on an individual, session-by-session basis across the six doses, providing mechanistic support for ketamine-psychotherapy pairings.

As well as being potentially more efficacious than stand-alone ketamine therapy, at least in the short term, ketamine-psychotherapy’s potential to yield benefits that are sustained psychologically rather than by repeated doses could translate to significant cost savings. However, the lack of head-to-head studies of ketamine plus or minus psychotherapy in depression currently precludes a definitive economic analysis, and there are no data yet on esketamine plus psychotherapy, although this is clearly a plausible approach.

## Conclusions

The story of ketamine from its development as a safer anaesthetic to now a breakthrough treatment in psychiatry has unfolded over 60 years through a combination of serendipity, rediscovery of early work, and the great clinical need for novel psychiatric treatments. This has been paralleled by a growing understanding of its psychopharmacology and brain actions, particularly relating to glutamate activation, network disruption, and neuroplasticity, which are at least partly common to the serotonergic psychedelics despite highly distinct pharmacology. Indeed, the recent renaissance of serotonergic psychedelics and its emphasis on psychotherapy challenges the early ways in which ketamine has been used as a stand-alone treatment of mental illness treatments as a sort of liquid ECT. Although recent results of ketamine-psychotherapy combinations are promising, audits of ongoing clinical usages and more systematic research are necessary to clarify the optimal ways in which ketamine should be used in psychiatry.
